# A common developmental plan for neocortical gene-expressing neurons in the pallium of the domestic chicken *Gallus gallus domesticus* and the Chinese softshell turtle* Pelodiscus sinensis*

**DOI:** 10.3389/fnana.2014.00020

**Published:** 2014-04-07

**Authors:** Ikuo K. Suzuki, Tatsumi Hirata

**Affiliations:** ^1^Division of Brain Function, National Institute of Genetics, Graduate University for Advanced Studies (Sokendai)Mishima, Japan; ^2^Institute of Interdisciplinary Research in Human and Molecular Biology, Université Libre de BruxellesBrussels, Belgium

**Keywords:** neocortex, neuron type, pallium, turtle, evolution

## Abstract

The six-layered neocortex is a unique characteristic of mammals and likely provides the neural basis of their sophisticated cognitive abilities. Although all mammalian species share the layered structure of the neocortex, the sauropsids exhibit an entirely different cytoarchitecture of the corresponding pallial region. Our previous gene expression study revealed that the chicken pallium possesses neural subtypes that express orthologs of layer-specific genes of the mammalian neocortex. To understand the evolutionary steps leading toward animal group-specific neuronal arrangements in the pallium in the course of amniote diversification, we examined expression patterns of the same orthologs and a few additional genes in the pallial development of the Chinese softshell turtle *Pelodiscus sinensis,* and compared these patterns to those of the chicken. Our analyses highlighted similarities in neuronal arrangements between the two species; the mammalian layer 5 marker orthologs are expressed in the medial domain and the layer 2/3 marker orthologs are expressed in the lateral domain in the pallia of both species. We hypothesize that the mediolateral arrangement of the neocortical layer-specific gene-expressing neurons originated in their common ancestor and is conserved among all sauropsid groups, whereas the neuronal arrangement within the pallium could have highly diversified independently in the mammalian lineage.

## INTRODUCTION

**FIGURE 1:**
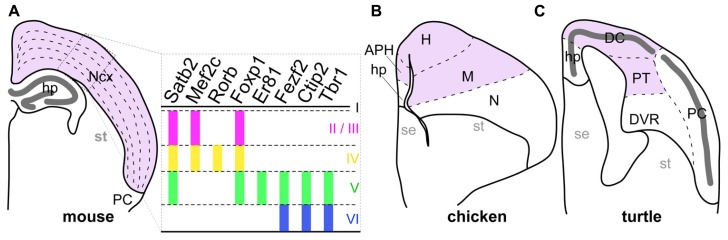
**Comparison of pallial structures among three amniotes.** The pallial subdivisions of the mouse **(A)**, chicken **(B)**, and turtle **(C)** are drawn based on their cytological characteristics. The mouse neocortex and its corresponding pallial regions in the chicken and turtle are colored in pink. The distinctive dense cellular layer in the turtle pallium is indicated by the thick dark-gray line. The right panel in **(A)** represents expression patterns of layer-specific transcription factor genes in the mammalian neocortex. Colored vertical bars indicate the layers in which each layer-specific marker gene is expressed. Abbreviations: APH, parahippocampal region; DC, dorsal cortex; DVR, dorsal ventricular ridge; H, hyperpallium; hp, hippocampus; M, mesopallium; N, nidopallium; Ncx, neocortex; PC, piriform cortex; PT, pallial thickening; se, septum; st, striatum.

Complex cognitive functions in mammalian species are essentially encoded in the neural circuits of the neocortex, a mammalian-specific structure characterized by tangential neuronal layers and located inside the pallium (the dorsal part of the telencephalon). In each layer, excitatory neurons with similar phenotypes are tangentially arranged. For example, extracortically projecting neurons reside in the deep layers 5 and 6, whereas the majority of intracortically connecting neurons are located in the more shallow layer 2/3 (Molyneaux et al., 2007). This laminar neuronal arrangement is basically shared by all studied mammalian species, including even the monotremes, and marsupials (Butler and Hodos, 2005). In contrast, the sauropsids, which is a group containing the currently living reptiles and birds, possess a totally different neuronal arrangement in the corresponding pallial region to the mammalian neocortex (**Figure 1**). This structural difference in neocortical regions has again raised the longstanding question of how mammals acquired the layered neocortex during evolution.

In mammalian neocortical development, layer-specific neuron subtypes differentiate from multipotent neural progenitors residing in the ventricular zone (VZ) of the pallium (Temple, 2001). Depending on the timing of their generation, newly generated neurons express transcription factors that provide layer-specific characteristics (Molyneaux et al., 2007; Greig et al., 2013). For example, layer 5 neurons generated earlier express *Fezf2* and its downstream target *Ctip2*, which are required and sufficient for their axons to project to extracortical targets (Arlotta et al., 2005; Chen et al., 2005, 2008; Molyneaux et al., 2005). In contrast, layer 2/3 neurons generated later express *Satb2*, a transcriptional repressor of *Ctip2*, and thereby project to intracortical targets by suppressing *Ctip2*-driven extracortical projections (Alcamo et al., 2008; Britanova et al., 2008). The fate-determining role of these transcription factors makes them ideal functional markers for layer-specific neuronal subtypes.

We, and other research groups, have recently found that the avian pallium houses neuron subtypes sharing the molecular expression, and axon projection patterns of layer-specific neuron subtypes in the mammalian neocortex (Dugas-Ford et al., 2012; Suzuki et al., 2012; Chen et al., 2013; Jarvis et al., 2013). In particular, the above-mentioned layer 5 transcription factors are expressed by neurons that occupy the medial domain in the pallium of chicks. On the other hand, the layer 2/3-specific transcription factors are expressed by neurons in the lateral domain (Suzuki and Hirata, 2012; Suzuki et al., 2012). These observations suggest that the pallia of sauropsids contain distinct neuronal populations that have characteristics similar to mammalian neocortical neurons, and raise an important question about the evolutionary changes of neuronal arrangements in the amniote pallia. What remained unclear was the generality of these findings among diverse amniote groups, because we only had limited knowledge of the neuronal arrangement in two distantly related animal groups: mammals and birds.

On the basis of our current understanding of amniote phylogeny, the lineages leading to the living mammals and the sauropsids diverged around 316 million years ago (Wang et al., 2013). Subsequently, the early sauropsids further diverged into several distinct reptilian groups, including the turtles. More recently, the birds originated from a reptilian group including the dinosaurs (Carroll, 1988; Kumar and Hedges, 1998; Hedges et al., 2006; Wang et al., 2013). Amniote pallia are histologically classified into three different types (**Figure 1**; Northcutt and Kaas, 1995; Medina and Reiner, 2000; Aboitiz and Zamorano, 2013). The first type has the six-layered cytoarchitecture only found in the mammalian neocortex (**Figure 1A**). The second has the domain architecture consisting of multiple nuclei commonly observed in birds (**Figure 1B**). The last is a simpler type found in non-avian reptiles such as lizards and turtles, in which a single neuronal layer spans the whole pallium (**Figure 1C**). Because it has the simplest cytoarchitecture, the pallium of the non-avian reptiles is considered as having retained the ancient state of the amniote common ancestor, from which the more complexly structured pallia in birds, and mammals have independently evolved (Marin-padilla, 1978; Medina and Reiner, 2000). However, despite its evolutionarily important position, gene expression in the reptilian pallium has been characterized in only a few studies (Dugas-Ford et al., 2012; Nomura et al., 2013a).

The current study describes comparative developmental expressions of gene orthologs of neocortical layer-specific transcription factors in the pallium of the Chinese softshell turtle *Pelodiscus sinensis*. We chose this turtle species because of the turtles’ phylogenetic position in the sauropsids, with a sister relationship to the birds (Kumazawa and Nishida, 1997; Iwabe et al., 2004; Wang et al., 2013). Even though small structural variations exist among reptile pallia (Aboitiz, 1999), this species has a pallium that represents the typical simple cytoarchitecture of non-avian reptiles well (**Figure 1C**). Furthermore, the commercial availability of fertilized eggs and accumulated knowledge about its embryonic development make this species an excellent model for detailed developmental research (Tokita and Kuratani, 2001; Nomura et al., 2013b). We examined the expression patterns of eight transcription factors whose layer-specific expression in the mammalian neocortex is implicated in the layer-specific characteristics of neurons (Molyneaux et al., 2007). We also advanced the characterization of gene expressions in the chick pallium beyond that achieved in previous studies by using other layer markers and differently staged embryos. On the basis of these results, we compare the differentiation and arrangement of neuron subtypes that express these genes in the pallia of different amniote groups, and discuss the evolutionary aspects of pallial organization.

## MATERIALS AND METHODS

### ANIMALS

Fertilized turtle and chicken eggs were purchased from local farmers, Daiwa Yoshoku (Oita, Japan) and Ohata Shaver (Shizuoka, Japan), respectively. We used 15 turtle and 15 chicken eggs for this study. The sexes of animals were uncertain. The day on which the eggs were transferred to 30°C (turtle) or 37°C (chicken) was designated as embryonic day 0 (E0). Developmental stages of the turtle and chicken embryos were defined according to previous studies (Hamburger and Hamilton, 1951; Tokita and Kuratani, 2001) and designated as TK and HH, respectively. All experimental protocols were approved by the Animal Committee of the National Institute of Genetics and carried out according to their guidelines.

### cDNA CLONING

The cDNA fragments coding for turtle *Satb2*, *Mef2c*, *Fezf2*, *Er81*, *Rorb*, and *Tbr1 *were amplified by RT-PCR with degenerate primers (**Table 1**) and subcloned into the plasmid vector pTA2 (TOYOBO, Tokyo, Japan). The cDNA fragment for chicken *Rorb* was similarly isolated using specific primers (**Table 1**). The other chicken genes used in the study were isolated in previous studies (Puelles et al., 2000; Sugiyama and Nakamura, 2003; Suzuki et al., 2012) and verified to contain the specified range of nucleotide sequences (**Table 1**) of the genes as annotated in the public databases.

**Table 1:** List of *in situ* probes used.

Name	Target	Primers for amplification of gene fragment
psSatb2	Turtle *Satb2*	5′-TGCCAGGAGTTTGGGAGATGG-3′ 5′-CTGTGTGCGRTTGAAWGCCAC-3′
psMef2c	Turtle *Mef2c*	5′-CCCACGCACTGAAGAAAAAT-3′ 5′-TTGTYGARATGGCTGATGGRT-3′
psER81	Turtle *Er81*	5′-CARGARACATGGCTTGCHGA-3′ 5′-ACTGGRTCRTGRTACTCCTG-3′
psFezf2	Turtle *Fezf2*	5′-GCGCAYTACAACCTSACSCGSC-3′ 5′-GGCTTCTTGTCRTTGTGSGT-3′
psTbr1	Turtle *Tbr1*	5′-CARGACCAGTTCGTSAGCAA-3′ 5′-CTGGAGTCGGACARGTCYTT-3′
psRorb	Turtle *Rorb*	5′-TCCARKCCAGACTGATCKGG-3′ 5′-GCCGMCTGCAGAAGTGYCTKG-3′
cSatb2	Chicken *Satb2* (Nucleotide 2302–2915 of XM_421919)	5′-ACCAGCACCCACAAGCTATCAACC-3′ 5′-ACTCCTCCTCATAGATCACATCCCTCTC-3′
cMef2c	Chicken *Mef2c* (Nucleotide 886–1583 of XM_001231661)	5′-CGTTGAGAAAGAAAGGACTTAATGG-3′ 5′-CCATCAGCCATCTCAACAACATATGGTAC-3′
cER81	Chicken *Er81* (Nucleotide 1868–2787 of NM_204917)	Gift from Dr. Nakamura Sugiyama and Nakamura (2003)
cFezf2	Chicken *Fezf2* (Nucleotide 280–1090 of XM_414411)	5′-CAAGAGCCTGGCCTTCTCCA-3′ 5′-TGAGCGTGGAGCTCCTGTTG-3′
cTbr1	Chicken *Tbr1* (Nucleotide 4–2132 of XM_003641638 and poly A tail)	Gift from Dr. Shimamura Puelles etal. (2000)
cRorb	Chicken *Rorb* (Nucleotide 682–1245 of XM_205093)	5′-GGTTTACAGCAACAGCATCAGCAAC-3′ 5′-GCTTGGAAGTGGTTTTGGTGAGAATGTG-3′

### DNA SEQUENCE ANALYSIS

Cloned genes were sequenced using a multi-capillary sequencer (ABI Prism 3130; Life Technologies, Carlsbad, CA, USA) and compared with the sequences of homologous genes obtained from the NCBI and ENSEMBL databases. The multiple sequence alignment was generated and edited using MEGA5 software (Tamura et al., 2011). Molecular phylogenetic trees were constructed by the neighbor joining method (Saitou and Nei, 1987) using MEGA5, and drawn with FigTree software (http://tree.bio.ed.ac.uk/software/figtree/).

### *IN SITU* HYBRIDIZATION

Brains of turtle and chick embryos were fixed with 4% paraformaldehyde in phosphate-buffered saline (PBS) overnight at 4°C and processed for coronal frozen sections. *In situ* hybridization was performed as previously described (Suzuki et al., 2012). Briefly, the sections were soaked with methanol at -30°C for 15 min, treated with 25 μg/ml proteinase K at room temperature for 5 min, and hybridized with 1 μg/mL DIG-labeled antisense RNA probes in hybridization buffer (50% formamide, 1 × Denhardt [Am-34-resco], 0.25 mg/mL RNA [Roche], 1 × PE, 100 μg/mL heparin, 0.1% Tween20, 0.75 M NaCl) at 60°C overnight. The probes were synthesized using the isolated cDNA clones (**Table 2**) as the templates. After washing, the hybridization signals were detected with anti-DIG antibody conjugated with alkaline phosphatase (Roche, Basel, Switzerland) and visualized with NBT/BCIP solution (Roche).

**Table 2:** Turtle cDNA fragments isolated.

Name	Accession No.	Length (bp)	Top blast hit
*Satb2*	AB689003 ENSPSIT00000020487	521	92% identical to chicken *Satb2* (XM_421919)
*Mef2c*	AB689005 ENSPSIT00000013823	601	93% identical to Green Anole *Mef2c *(XM_003216358)
*Er81*	AB689006 ENSPSIT00000009495	547	93% identical to chicken *Er81* (NM_204917)
*Fezf2*	AB689007 ENSPSIT00000010810	387	90% identical to chicken *Fezf2* (XM_414411)
*Tbr1*	AB689004 ENSPSIT00000004703	532	87% identical to chicken *Tbr1* (XR_026840)
*Rorb*	AB689002 ENSPSIT00000006123	319	90% identical to Green Anole *Rorb *(XM_003216536)

### IMMUNOHISTOCHEMISTRY

Brain sections were prepared as described for *in situ* hybridization. The sections were washed with TBST (10 mM Tris pH7.4, 150 mM NaCl, 0.1% Tween20), permeablized with 100% methanol at -30°C for 15 min, and then reacted with the primary antibodies at 4°C overnight. The following antibodies were used: mouse anti-Cadherin7 antibody [CCD7-1; Developmental Studies Hybridoma Bank (DSHB), Iowa, IA, USA], rat anti-*Ctip2* antibody (ab18465; Abcam, Cambridge, UK), rabbit anti-Foxp1 antibody (ab16645; Abcam), mouse anti-β (III)-tubulin antibody, TUJ1 (MMS-435P; Covance, Princeton, NJ, USA), and rabbit anti-phospho-Histone H3 antibody (06-570; Millipore, Billerica, MA, USA), After washing with TBST, the sections were stained with anti-mouse IgG antibody conjugated with Alexa Fluor 488 (A11029; Life Technologies), anti-rat IgG antibody conjugated with Alexa Fluor 488 (A11006; Life Technologies) or anti-rabbit IgG antibody conjugated with Cy3 (711-165-152; Jackson ImmunoResearch, West Grove, PA, USA). They were then washed with TBST again, and coverslipped with 90% glycerol in PBS.

### IMAGING

Bright-field and fluorescence images were captured using a fluorescent microscope (Axioplan2; Zeiss, Jena, Germany) using a CCD camera (DP71; Olympus, Tokyo, Japan). The brightness and contrast of images were adjusted using Adobe Photoshop CS4 software (Adobe Systems; San Jose, CA, USA).

## RESULTS

### PALLIAL SUBDIVISIONS IN THE TURTLE

**FIGURE 2:**
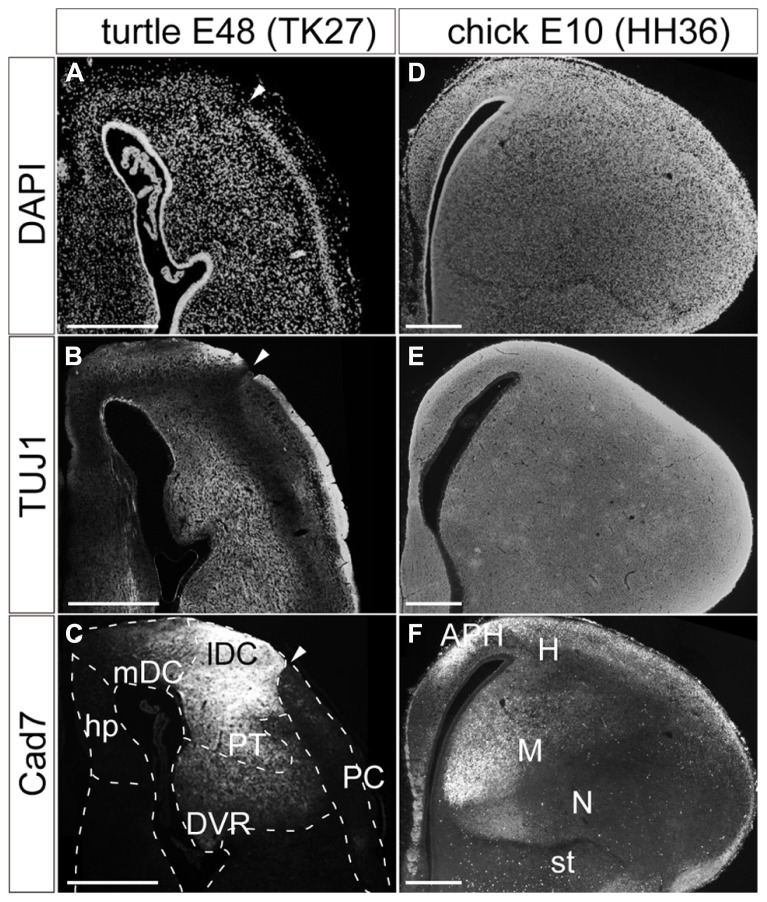
**Pallial subdivisions of the turtle and chick.** Coronal sections of the turtle **(A**–**C)** and chick **(D**–**F)** pallia at E48, and at E10, respectively, in which the mediolateral axis runs left to right, and the dorsoventral axis runs top to bottom. **(A**,**D)** Nuclear staining by DAPI. **(B**,**E)** Immunostaining with TUJ1 antibody strongly labels axons in the turtle and chick pallia. **(C,F)** Immunostaining for Cad7. Note the strong specific labeling of the lateral part but not the medial part of the turtle DC and the chick mesopallium. White arrowheads indicate the boundary between the DC and PC in the turtle pallium. Scale bars: 250 μm. Abbreviations: Cad, cadherin; DVR, dorsal ventricular ridge; hp, hippocampus; lDC, lateral part of the dorsal cortex; mDC, medial part of the dorsal cortex; PC, piriform cortex; PT, pallial thickening.

The matured turtle pallium has been cytologically subdivided into several domains in the previous literature (Johnston, 1915; Powers and Reiner, 1980; Goffinet, 1983). Briefly, the trilaminar domain covers the superficial part, and is further subdivided into the hippocampus, dorsal cortex (DC), and piriform cortex (PC) along the mediolateral axis (**Figure 1C**). These three subdivisions commonly have the cytological organization that contains a single layer of pyramidal neurons (**Figure 2A**). This layer is sandwiched between the exterior, axon-dense, and interior, axon-sparse, layers (**Figure 2B**) that also contain scattered interneurons (Blanton and Kriegstein, 1991). The pyramidal neurons in the single packed layer possibly consist of multiple neuronal populations that have distinct connection targets and molecular expressions, as shown in some other reptile species (Martínez-Marcos et al., 1999; Nomura et al., 2013a). Underneath the trilaminar domain are the nuclear domains that consist of the pallial thickening (PT) and dorsal ventricular ridge (DVR). The former is continuously extended ventrally from the DC, and the latter protrudes into the lateral ventricle from the most ventral part of the pallium (**Figure 1C**). Among these subdivisions of the turtle pallium, the DC, and PT are accepted as the homolog of the mammalian neocortex because of their shared features, including the expression of *Emx1,* and reciprocal connections with the thalamus (Heller and Ulinski, 1987; Fernandez et al., 1998; Reiner, 2000).

In contrast, the chick pallium does not have an apparent cellular layer. Until the nuclear structure manifests with clustered neurons by the end of the embryonic stages, neurons are more or less evenly scattered across the pallium (**Figures 2D,E**). The protrudent DVR in the chick pallium is anatomically subdivided into the dorsal mesopallium and ventral nidopallium. The former division and the hyperpallium are accepted as the *Emx1*-positive field homologous to the mammalian neocortex (Fernandez et al., 1998).

Expression of cadherin proteins, in particular Cad7, has been used to define the avian pallial subdivisions (Redies et al., 2001). In the developing chick pallium, Cad7 labeled the parahippocampal region (APH) and other restricted parts of the pallial divisions (**Figure 2F**). Of note is that its restricted expression further subdivides the pallium. For example, the medial parts of the mesopallium or nidopallium are compartmentalized by strong Cad7 expression. Likewise, the same antibody against Cad7 supported further subdivisions of the turtle pallium (**Figure 2C**). The protein was strongly expressed in parts of the DC and PT. In the DC, it was more strongly expressed in the lateral domain, clearly delineating the lateral from the medial DC. Thus, in a molecular context, the turtle DC seems divisible into medial and lateral compartments, although the two domains appeared cytologically homogeneous and continuous.

### CHARACTERIZATION OF LAYER-SPECIFIC MARKER ORTHOLOGS OF THE TURTLE

**FIGURE 3:**
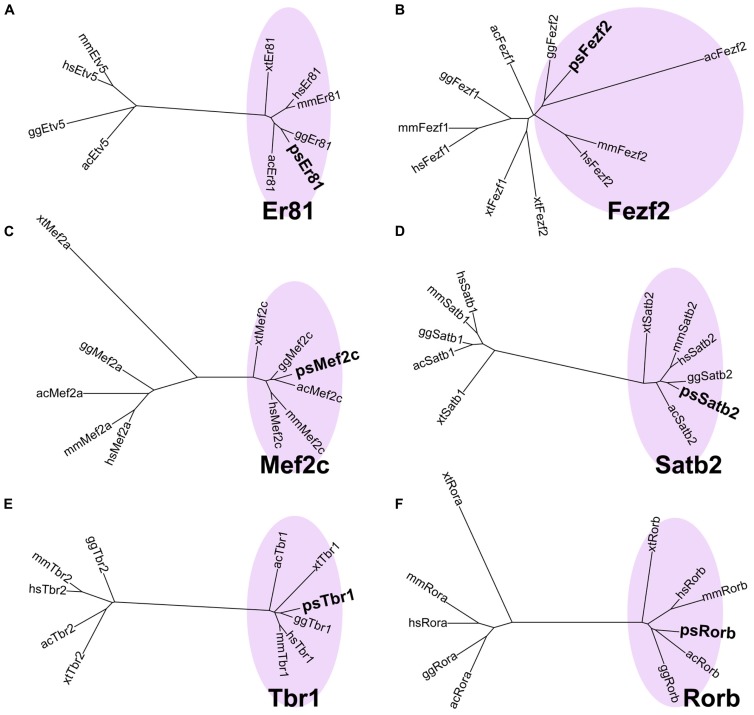
**Verification of layer-specific marker orthologs in the turtle.** Molecular phylogenetic trees of **(A)** *Er81*, **(B)** *Fezf2*, **(C)** *Mef2c*, **(D)** *Satb2*, **(E)** *Tbr1*, and **(F)** *Rorb* cDNAs, constructed by the neighbor joining (NJ) method. Turtle cDNAs cloned in this study are indicated in boldface. In all six phylogenetic relationships, the turtle cDNAs are clustered with the orthologous genes (pink circled area) and separated from the paralogous genes. Abbreviations: ac, *Anolis carolinensis* (Green anole); gg, *Gallus gallus* (Chicken); hs, *Homo sapiens* (Human); mm, *Mus musculus* (Mouse); ps, *Pelodiscus sinensis* (Turtle); xt, *Xenopus tropicalis* (Frog).

When we first cloned turtle cDNAs, the genomic sequence of the turtle was not available. Thus, we amplified partial fragments of orthologous cDNAs of the six mammalian layer-specific markers, *Satb2*, *Mef2c*, *Rorb*, *Er81*, *Fezf2*, and *Tbr1 *(**Figure 3**), by reverse transcriptase-polymerase chain reaction (RT-PCR) using degenerate primers designed for the consensus sequences deduced from amniote genes (**Table 1**). To confirm whether the cloned turtle cDNAs indeed encoded the designated genes, we analyzed the cDNA sequences using BLASTN, and the NCBI mRNA database (Reference RNA sequences) to identify the most closely related sequences. This survey found the highest similarity for each clone to the expected ortholog of the chicken (*Gallus gallus domesticus*) or the green anole (*Anolis carolinensis*; **Table 2**). We next constructed phylogenic trees for these six isolated clones by incorporating sequence data from the human (*Homo sapiens*), mouse (*Mus musculus*), chicken, green anole, and frog (*Xenopus tropicalis*; **Figure 3**). The cDNA clones from the turtle were closely clustered with the designated orthologous members of these species and not with paralogs, confirming that they were indeed the turtle orthologs of the mammalian layer-specific marker genes (**Figure 3**). After the draft genome sequence of Chinese soft shell turtle was released recently (Wang et al., 2013), we confirmed that all the cloned cDNA sequences corresponded to those of the recorded transcripts in the database.

### DEVELOPMENTAL EXPRESSION OF LAYER-SPECIFIC MARKERS IN THE TURTLE PALLIUM

**FIGURE 4:**
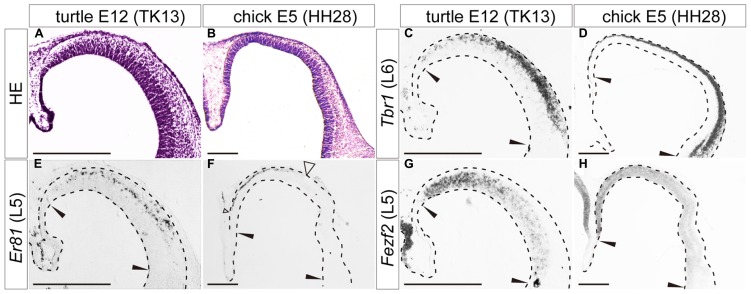
**Expression of layer-specific marker genes in the turtle and chick pallia at the early neurogenetic stage.** Coronal sections of E12 turtle **(A,C,E,G)** and E5 chick **(B,D,F,H)** pallia, in which the mediolateral axis runs left to right and the dorsoventral axis runs top to bottom. **(A,B)** Hematoxylin and eosin (HE) staining of the pallium. **(C,D)** Expression patterns of *Tbr1*, a layer 6 (L6) marker. **(E,F)** Expression patterns of *Er81*, a layer 5 (L5) marker. An open arrowhead in **(F)** indicates the lateral edge of the *Er81*-expression domain in the chick pallium. **(G,H)** Expression patterns of *Fezf2*, a layer 5 (L5) marker. Black arrowheads in each panel indicate the medial and lateral ends of the pallium. Scale bars: 250 μm.

**FIGURE 5:**
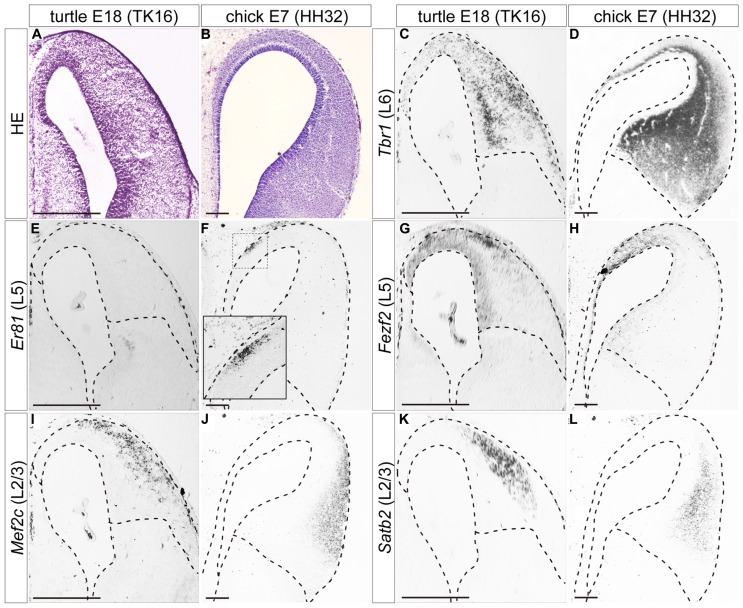
**Expressions of layer-specific marker genes in the turtle and chick pallia at the mid-neurogenetic stage.** Coronal sections of E18 turtle **(A,C,E,G,I,K)** and E7 chick **(B,D,F,H,J,L)** pallia, in which the mediolateral axis runs left to right and the dorsoventral axis runs top to bottom. **(A,B)** HE staining of the pallium. **(C)** *Tbr1* expression exhibits a medial-low, lateral-high gradient across the whole pallium of the turtle. **(D)** *Tbr1* expression covers the whole chick pallium except for the marginal and VZs. **(E,F)** *Er81* is not expressed in the turtle pallium **(E)**, but is expressed specifically in the medial part of the chick pallium **(F)**. The inset is an enlargement of the rectangular box in the medial region. **(G,H)** *Fezf2* expression exhibits a medial-high, lateral-low gradient in the VZ and differentiated neurons in both species. **(I**–**L)** Two layer 2/3 (L2/3) marker genes, *Mef2c* and *Satb2*, are expressed in the lateral region of the turtle and chick pallia. Scale bars: 250 μm.

**FIGURE 6:**
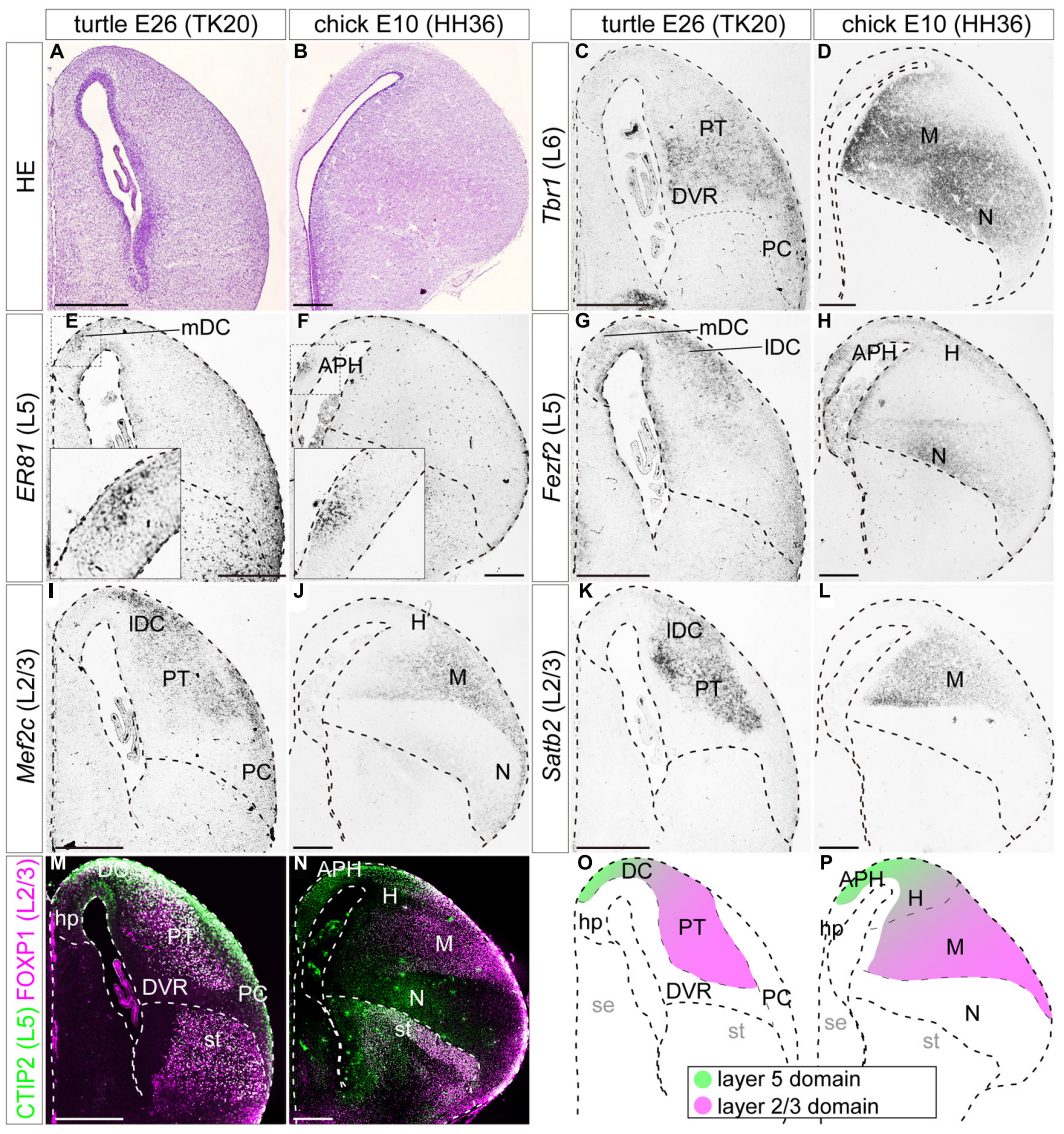
**Expressions of layer-specific marker genes in the turtle and chick pallia at the terminal neurogenetic stage.** Coronal sections of E26 turtle **(A,C,E,G,I,K,M)** and E10 chick **(B,D,F,H,J,L,N)** pallia, in which the mediolateral axis runs left to right and the dorsoventral axis runs top to bottom. **(A,B)** Hematoxylin and eosin (HE) staining of the pallium. **(C,D**) *Tbr1* is enriched in the ventrolateral side of the turtle and chick pallia. **(E,F)** *Er81* is specifically localized in the small medial region of the turtle and chick pallia. The insets are enlargements of the dashed squares. **(G)** *Fezf2* expression is widespread in the turtle DC, but does not expand to the other regions such as the PT, DVR, or PC. **(H)** In the chick, *Fezf2 *expression is concentrated in the APH and the ventral nidopallium, and weakly detected in the hyperpallium. *Fezf2* and *Er81* expressions are overlapped in the medial part of the pallium in both species. **(I**–**L)** *Mef2c* and *Satb2* expressions are localized in the lateral part of the turtle and chick pallia. **(M,N)** Double immunostaining for *Ctip2* (green) and Foxp1 (magenta). *Ctip2* signals are broadly distributed across the turtle pallium including the DC, PT, and PC **(M)**, whereas they are more specifically localized in the medial part of the chick pallium **(N)**. Foxp1 is expressed in the turtle PT and the chick mesopallium. Scale bars: 250 μm. **(O,P)** Schematic illustrations of the turtle and chick pallial subdivisions based on the expression patterns of seven layer-specific marker genes. Tbr1-expressing domain is not illustrated for simplicity. The distribution of *Tbr1*-expressing neurons is discussed later in the discussion section. Abbreviations: APH, parahippocampal region; DC, dorsal cortex; DVR, dorsal ventricular ridge; H, hyperpallium; hp, hippocampus (hippocampal homolog); lDC, lateral part of dorsal cortex; M, mesopallium; mDC, medial part of dorsal cortex; N, nidopallium; PC, piriform cortex; PT, pallial thickening; se, septum; st, striatum.

**FIGURE 7:**
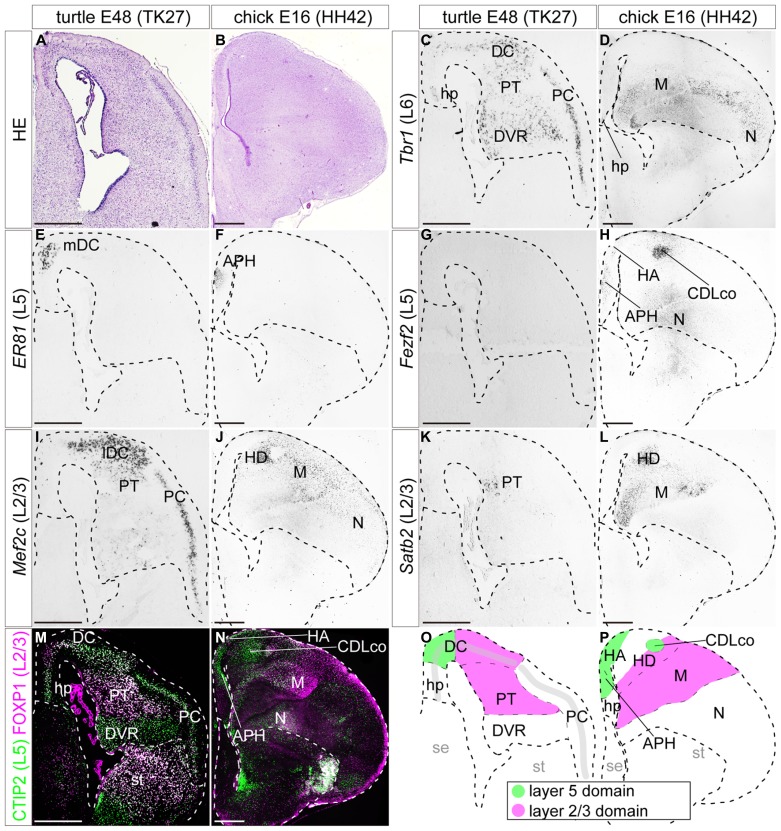
**Expression of layer specific marker genes in the turtle and chick pallia at the maturation stage.** Coronal sections of E48 turtle **(A,C,E,G,I,K,M)** and E16 chick **(B,D,F,H,J,L,N)** pallia, in which the mediolateral axis runs left to right and the dorsoventral axis runs top to bottom. **(A,B)** Hematoxylin and eosin (HE) staining of the pallium. **(C)** *Tbr1* is expressed in virtually all the regions of the turtle pallium. **(D)** In the chick pallium, *Tbr1* expression is detected in the ventrolateral regions, the mesopallium and the nidopallium. **(E,F)** *Er81* expression is exclusively restricted to the small medial regions of the turtle and chick pallia. **(G)** *Fezf2* is not expressed in the turtle at this stage. **(H)** In the chick, *Fezf2* is weakly expressed in the medial regions of the pallium in addition to the strong expression in the CDLco and nidopallium. **(I**–**L)** *Mef2c* and *Satb2* are expressed in the lateral part of the turtle and chick pallia. *Mef2c* is more marginally expressed than *Satb2*. **(M,N)** Double immunostaining for *Ctip2* (green) and Foxp1 (magenta). *Ctip2* is expressed in virtually all areas in the turtle pallium **(M)**, but is specifically confined to the medial part in the chick pallium **(N)**. The expression of Foxp1 is confined to the lateral part of the turtle and chick pallia. Scale bars: 250 μm. **(O,P)** Schematic illustrations of the turtle and chick pallial subdivisions based on the expression patterns of the seven layer-specific marker genes. Abbreviations: APH, parahippocampal region; CDLco, core nucleus of caudodorsolateral pallium; DC, dorsal cortex; DVR, dorsal ventricular ridge; H, hyperpallium; HA, apical part of the hyperpallium; HD, densocellular part of the hyperpallium; hp, hippocampus (hippocampal homolog); lDC, lateral part of the dorsal cortex; M, mesopallium; mDC, medial part of the dorsal cortex; N, nidopallium; PC, piriform cortex; PT, pallial thickening; se, septum; st, striatum.

We analyzed the expression patterns of the layer-specific markers in the developing turtle pallium and compared them with those in the chick pallium. The developmental stages of the turtle embryo were empirically matched with the chicken stages that display similar histological characteristics in the pallium, (**Figures 4A,B**, **5A,B**, **6A,B**, and **7A,B**), such as the thickness of the VZ and the density of mitotic cells in the VZ. Correlation of the stages was also confirmed as appropriate based on the maximally shared transcriptome of the whole embryos (Wang et al., 2013). In addition to the six newly cloned turtle orthologs of layer-specific markers, we also used antibody markers for *Foxp1*, and *Ctip2*, which specifically recognize the turtle and chicken proteins (Suzuki et al., 2012). Among the eight layer-specific markers, we successfully detected the expression of seven marker genes: *Tbr1*, *Er81*, *Fezf2*, *Mef2c*, *Satb2*, *Foxp1*, and *Ctip2*, in the developing turtle pallium. Only the expression of *Rorb*, a layer 4 marker, was undetectable in the turtle pallium at all developmental stages examined, although this gene was weakly expressed in the chick mesopallium and nidopallium at E16 (data not shown).

When neurogenesis had just started in the pallium, at E12 in turtles and E5 in chicks, the layer 6 marker *Tbr1*, and the layer 5 marker *Er81* were already expressed broadly by differentiated neurons in the pallium (**Figures 4C–F**). These expressions were not limited to the medial domain but stretched across the pallium, although *Er81* was medially enriched in the chick pallium. Another layer 5 marker, *Fezf2*, was not expressed by postmitotic neurons in the pallium, but only detected in neural progenitors of the VZ, making a medial-high to lateral-low gradient, in both turtles and chicks (**Figures 4G,H**). None of the three layer 2/3 markers were detected in the pallium at these early neurogenetic stages.

In slightly advanced mid-neurogenetic stages, E18 in turtles, and E7 in chicks, the layer 6 marker* Tbr1* was expressed by neurons in virtually all areas of the pallium in both species (**Figures 5C,D**). The expression level was more intense in the lateral side than in the medial side. The expression of the layer 5 marker *Er81* was not detected in the turtle pallium at this stage (**Figure 5E**), but was detected in a small confined domain in the medial, superficial part of the chick pallium (**Figure 5F**) as reported previously (Nomura et al., 2008; Suzuki et al., 2012). At these stages, the expression of the layer 5 marker *Fezf2* was no longer restricted to the VZ, but also expanded to include medially scattered neurons in the turtle and chick pallia. *Fezf2* expression covered the *Er81*-expressing region in the chick pallium (**Figures 5G,H**). By these stages, two layer 2/3 marker genes, *Satb2*, and *Mef2c*, started to be expressed in the lateral part of the pallium in both turtles and chicks (**Figures 5I–L**). Although the expression domains of the two genes largely overlapped, a closer look revealed that *Mef2c* expression slightly shifted to the marginal surface when compared to *Satb2* expression in both species. The lateral domain commonly marked with the two layer 2/3 genes was not completely separated from the medial domain marked with the layer 5 marker *Fezf2*, and there was substantial overlap between the *Fezf2*- and *Satb2/Mef2c*-positive domains in both turtles and chicks.

When neurogenesis is almost terminated, at E26 in turtles, and E10 in chicks, the layer 6 marker *Tbr1* was still broadly expressed in the large, lateral part of the pallium in turtles and chicks (**Figures 6C,D**). The layer 5 marker *Er81* was expressed in a very small restricted domain in the medial side of the pallium in both species (**Figures 6E,F**). These *Er81*-expressing medial domains of the two species were seemingly equivalent, but technically have been defined differently, as the turtle mDC and the chick APH. At these stages, the layer 5 marker *Fezf2* reduced its expression in the VZ and was mainly expressed by differentiated neurons (**Figures 6G,H**). These *Fezf2*-expressing neurons showed a more confined distribution compared to earlier stages, but still covered a larger area than the *Er81*-expressing domain. More specifically, in the chick, signals for *Fezf2* were most strongly concentrated in the *Er81*-expressing APH, and further spread weakly over the laterally positioned hyperpallium, and nidopallium. In the turtle, the signals spread more widely over the whole region of the DC, including into the *Er81*-expressing medial domain. From these stages, the antibody markers started to show a specific labeling pattern (**Figures 6M,N**). In the chick pallium, as reported in the previous study (Suzuki et al., 2012), the layer 5 marker *Ctip2* protein was expressed mainly in the APH and the dorsal part of the hyperpallium, which is presumably the future apical part of the hyper pallium (HA; green in **Figure 6N**). The expression pattern was very similar to that of *Fezf2*, which is the transcriptional activator of *Ctip2* in mammals (Chen et al., 2005, 2008; Molyneaux et al., 2005). In the turtle, *Ctip2* was expressed more widely than *Fezf2,* and covered not only the DC but also other pallial areas, namely the PT and PC (green in **Figure 6M**). At these late developmental stages, three layer 2/3 marker genes, *Mef2c*, *Satb2*, and *Foxp1*, were all expressed in the lateral side of the pallium in turtles and chicks (**Figures 6I–N**). More specifically, in the turtle, the lateral compartment of the DC and PT were intensely labeled by all three markers, whereas the mesopallium and the neighboring densocellular part of the hyperpallium (HD) were labeled in the chick. In summary, the expression patterns of each layer-specific marker in the turtle and chick pallium resembled each other. A common trend observed in both species was that the layer 5 and the layer 2/3 markers were segregated in the medial and lateral domains, with a small overlap in the dorsal region of the pallium (**Figures 6O,P**).

By the end of embryonic development, at E48 in turtles and E16 in chicks, many of the marker genes had restricted their expression domains. In the turtle, the expression of layer 6 marker* Tbr1* was weakened, displaying a medial-low, lateral-high gradient across the whole pallium (**Figure 7C**). In the chick, *Tbr1* only marked the ventral region of the pallium, including the mesopallium and nidopallium (**Figure 7D**). The expression of the layer 5 marker *Er81* continued to be confined to the most medial compartment of the DC in the turtle and the APH in the chick (**Figures 7E,F**). The expression of the layer 5 marker *Fezf2* became undetectable in the turtle pallium (**Figure 7G**). In the chick, *Fezf2* expression was still weakly detected in the APH and HA, and in addition, was strongly expressed in the core nucleus of the caudodorsolateral pallium (CDLco) and nidopallium (**Figure 7H**). The layer 5 marker *Ctip2* was still widely expressed in the turtle pallium (**Figure 7M**), whereas in the chick pallium, the expression was restricted to the *Fezf2*-positive domains (**Figure 7N**). The three layer 2/3 marker genes, *Mef2c*, *Satb2*, and *Foxp1*, continued to be expressed with significant overlap in the lateral domain of the pallium, the lateral part of DC (lDC), and PT in turtles, and the HD and mesopallium in chicks (**Figures 6I–N**). Similar to the slightly heterogeneous expressions of these genes reported in the chick (Suzuki et al., 2012), the spatial expression patterns of the layer 2/3 genes were also substantially different from each other in turtles; the expression of *Mef2c* was mostly concentrated in the neuronal layer in the DC and PC, but not significantly detected in the internal nuclei, PT, and DVR (**Figure 7I**). In contrast, the expressions of *Satb2* and Foxp1 were mainly detected in the PT (**Figures 7K,M**). Lastly, in these relatively mature stages, the overall expression domains of the layer 5 and layer 2/3 markers were more clearly segregated mediolaterally in the pallium of both turtles and chicks. The boundaries between the layer 5 and layer 2/3 domains were positioned in the DC in the turtle, and in the hyperpallium in the chick (**Figures 7O,P**).

### SIMILARITY OF THE NEUROGENETIC PATTERNS IN THE TURTLE AND CHICK PALLIA

**FIGURE 8:**
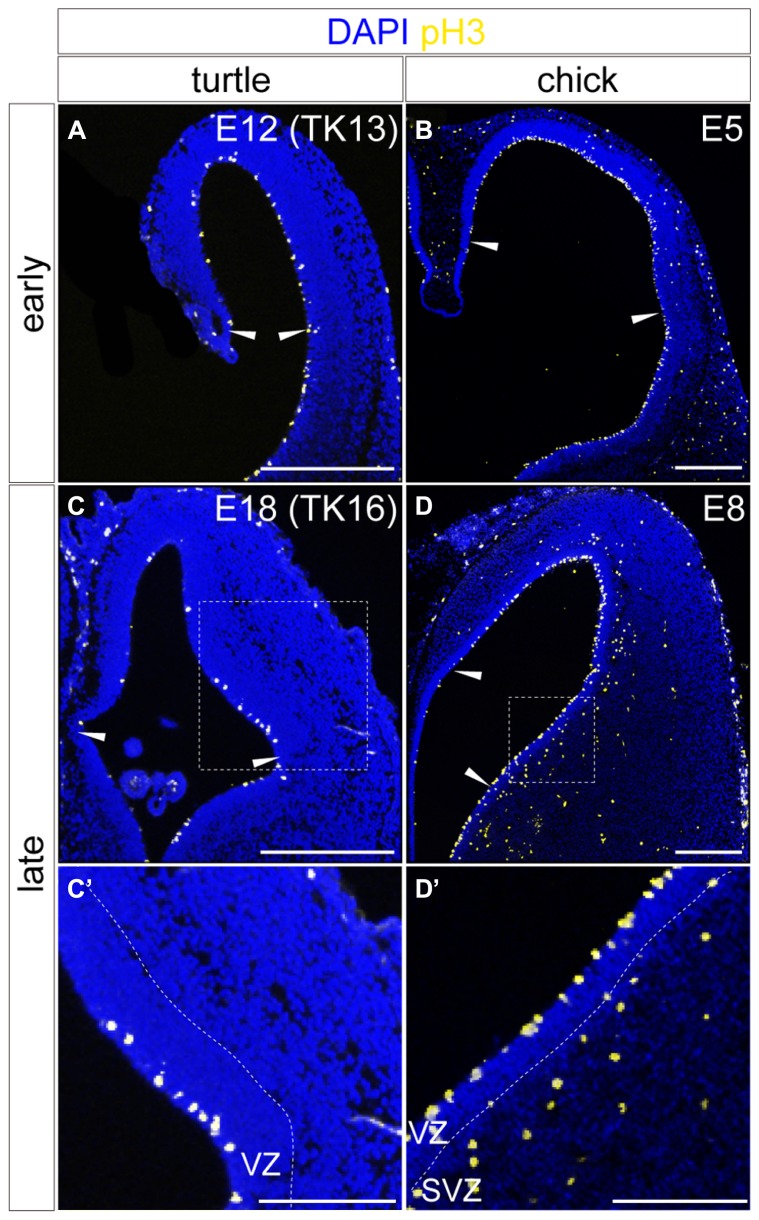
**Spatial distribution of mitotic cells in the developing turtle and chick pallia.** Mitotic cells are labeled by anti-pH3 antibody in the turtle **(A,C)** and chick **(B,D)** pallia at early **(A,B)** and late **(C,D)** developmental stages; the mediolateral axis runs left to right and the dorsoventral axis runs top to bottom. Arrowheads indicate the medial and lateral borders of the pallium. White dotted insets in **(C)** and **(D)** are magnified in **(C’,D’)**. Scale bars: 250 μm **(A**–**D)**, 125 μm **(C’,D’)**.

The spatiotemporal control of neurogenetic activities is a key factor that eventually creates distinct neuronal arrangements and morphological characteristics in different animal pallia (Suzuki et al., 2012; Aboitiz and Zamorano, 2013). We therefore examined neurogenetic activities in the turtle pallium and compared them to those in the chick. In the early stage of neurogenesis at E12, the mitotically active cells labeled by anti-phospho-histone H3 antibody were evenly distributed in the VZ throughout the pallium of the turtle, as observed in the chick at E5 (**Figures 8A,B**). In contrast, in the later stage at E18, the mitotically active cells were more abundantly observed in the lateral than in the medial side of the turtle pallium (**Figure 8C**). This laterally biased distribution of mitotic cells in the late phase of neurogenesis resembled that in the chick pallium at E8 (**Figure 8D**), in which it causes the late lateral expansion of the neuron population (Nomura et al., 2008; Suzuki et al., 2012). One minor difference between turtle and chick neurogenesis was that subventricular mitosis was lacking in the turtle pallium but existed in the chick pallium (**Figures 8C’,D’**), as reported in a previous study (Cheung et al., 2007).

## DISCUSSION

We studied turtle pallial development based on the expression patterns of eight layer-specific transcription factor genes, *Tbr1*, *Er81*, *Fezf2*, *Ctip2*, *Rorb*, *Foxp1*, *Mef2c*, and *Satb2*, and compared them with those in the chick pallium. We selected these genes not only as markers, but as potential determinants of neuronal fates, because many of them specified neuronal characteristics such as connection patterns in the rodent neocortex (Greig et al., 2013). The comparison indicated that the turtle and chick fundamentally share the spatial arrangement of molecularly defined neuron subtypes in the pallium. In both species, the layer 5, and layer 2/3 layer-specific genes are expressed by neurons in the medial and lateral domains of the pallium, respectively. Furthermore, the turtle and chicken also shared spatiotemporal patterns of embryonic neurogenesis, leading to the expansion of late-born neurons in the lateral domain. Taken together, these results suggest that the developmental scheme of the turtle pallium is basically similar to that of the chick pallium, and distinct from that of the mammalian neocortex.

### EXPRESSION OF LAYER-SPECIFIC TRANSCRIPTION FACTOR GENES

#### Layer 6 marker, Tbr1

The layer 6 marker *Tbr1* gene was broadly expressed across the pallium in both turtles and chicks. This expression extensively overlapped with that of layer 2/3 markers in the lateral part of the pallium. In certain developmental stages such as E18 in turtles and E7 in chicks (**Figure 5**), the layer 2/3 genes were more superficially expressed in the thin lateral domain, whereas *Tbr1* was more widely expressed, as well as deeply, in the vicinity of the VZ. This configuration may give an impression of recapitulating the mammalian inside-out arrangement of layer 6 and 2/3 neurons. However, we consider it premature to discuss this possibility for the following reasons. First, we only analyzed one layer 6 marker in the analyses. Our and others’ previous attempts to detect other layer 6 marker genes, such as Sox5, Foxp2, and Grg4, have failed to show their specific expressions in the chick pallium (Teramitsu et al., 2004; I.K.S. unpublished observation). Second, *Tbr1* is not an exclusive marker for the neocortical layer 6, but is additionally expressed in other areas such as the neocortical layer 2/3, the hippocampus and the olfactory cortex (Hevner et al., 2001; Englund et al., 2005). Therefore, we cannot be certain that the wide expression of *Tbr1* in the turtle and chicken pallia in fact recapitulates the expression as the layer 6 marker. Finally, newly born neurons migrate roughly outside-in, but never inside-out, in the pallia of birds, and reptiles. Taken together, we consider the analogous counterparts of mammalian layer 6 neurons in chicks and turtles an open question.

#### Layer 5 markers, Er81, Fezf2, and Ctip2

We examined three markers expressed in layer 5 of the mammalian neocortex (Hashimoto et al., 2000; Matsuo-Takasaki et al., 2000; Arlotta et al., 2005; Yoneshima et al., 2006), *Er81*, *Fezf2*, and *Ctip2*. Among the three genes at least, *Fezf2*, and its downstream target, *Ctip2*, are shown to be functionally crucial for providing the characteristics to mammalian layer 5 neocortical neurons and enabling them to project their axons to the brainstem (Chen et al., 2005, 2008; Molyneaux et al., 2005). All of these layer 5 genes were consistently expressed in the medial part of the turtle and chick pallium in an overlapping manner, leading to the assumption that these medial neurons have similar characteristics to the mammalian layer 5 neurons. Compared with the completely overlapping expressions of *Fezf2 *and *Ctip2* in the mammalian, and chick pallia, *Ctip2* was expressed more widely than *Fezf2 *in the turtle pallium. Therefore, in the turtle, the transcription of *Ctip2 *may be controlled by another regulator in addition to the upstream activator *Fezf2*. The expression patterns of these layer 5 marker genes observed in this study are consistent with the gene expression data recently reported in multiple reptile (Dugas-Ford et al., 2012; Nomura et al., 2013a) and avian species (Chen et al., 2013; Jarvis et al., 2013).

#### Expression of layer 4 marker, Rorb

Although we could not detect a specific expression of *Rorb*, recent reports have revealed its expression in the chicken and turtle pallia (Atoji and Karim, 2012; Dugas-Ford et al., 2012; Chen et al., 2013; Jarvis et al., 2013). Furthermore, Eag2, another layer 4 marker is also expressed in the same domains. Their shared expression suggests that the caudolateral DC of turtles and the part of hyperpallium and nidopallium entail neurons analogous to mammalian layer 4 neurons.

#### Expression of layer 2/3 markers, Foxp1, Mef2c, and Satb2

For this category, we examined three markers, *Foxp1*, *Mef2c*, and *Satb2*, which are strongly expressed in layer 2/3 of the mammalian neocortex (Leifer et al., 1993; Ferland et al., 2003; Zhong et al., 2004; Britanova et al., 2005; Szemes et al., 2006; Hisaoka et al., 2010). Among these genes, *Satb2 *is critically important for providing intracortical projection identity to late-generated layer 2/3 neurons in mice (Alcamo et al., 2008; Britanova et al., 2008). All of the layer 2/3 marker genes exhibited similar overlapping expressions in the lateral part of the turtle and chick pallia. Although the slight variation in expression patterns of individual genes suggested some heterogeneity in the neuronal population as seen in layer 2/3 of mammalian neocortex, their consistent expressions in the same domain suggest that these neurons in the lateral domain are molecularly analogous to mammalian layer 2/3 neurons.

### COMPARISON OF ARRANGEMENTS OF MOLECULARLY DEFINED NEURAL POPULATIONS IN THE PALLIA OF AMNIOTES

In both turtle and chick pallia, the layer 5 and layer 2/3 marker-expressing neurons were segregated into the medial and lateral domains, respectively. Thus, the arrangements of these molecularly defined neuronal subtypes in the pallia are highly similar between the two distant sauropsid species, suggesting a common developmental plan for pallial neurogenesis in the sauropsids. More specifically, layer 5 marker-expressing neurons are localized in the medial DC of the turtle, and the APH, and apical part of the hyper pallium (HA) of the chick. In contrast, layer 2/3 marker-expressing neurons are localized in the lateral DC, and PT of the turtle, and in the densocellular part of the hyperpallium (HD), and mesopallium of the chick. Although the turtle DC and the chick hyperpallium have seemingly homogeneous histological features, they are actually subdivided into medial and lateral domains based on gene expression. This is also consistent with previous reports that demonstrate functional specialization of the medial and lateral domains of the turtle DC (Heller and Ulinski, 1987; Ulinski, 2007) and the chick hyperpallium (Butler and Hodos, 2005; Jarvis et al., 2005).

Given that these molecularly defined neuronal populations were sauropsid counterparts of mammalian layer-specific neurons, how can we reconcile the differences in the neuronal arrangement of sauropsids and mammals? We recently proposed that the spatiotemporal pattern of neurogenetic activities differentiates the chick pallium from that of mammals (Suzuki and Hirata, 2012, 2013; Suzuki et al., 2012). This hypothesis is based on our observation that chick pallial neural progenitors produce layer 5 and layer 2/3 marker-expressing neurons in the same temporal sequence as that of mammalian progenitors in isolated culture conditions. This neocortex-like neurogenetic potential in chick neural progenitors is partially suppressed spatiotemporally in the chick pallium *in vivo*. Namely, neural progenitors on the medial side of the chick pallium precociously terminate neurogenesis before producing the late-born layer 2/3 marker-expressing neurons, the medial domain is thereby dominated by the early born layer 5 marker-expressing neurons. On the other hand, neural progenitors on the lateral side greatly expand their neurogenetic activities in the late developmental stage, and thereby accumulate the later-born layer 2/3 marker-expressing neurons in the lateral domain (Suzuki et al., 2012). The conserved spatiotemporally biased neurogenetic activities in chick and turtle pallia (**Figure 8**) suggest that their common neurogenetic pattern creates similar medio-laterally separated neuronal arrangements in their pallia.

The mechanisms underlying this spatiotemporally biased neurogenesis remain unclear, but extrinsic factors in the *in vivo* environment are implicated (Suzuki et al., 2012; Suzuki and Hirata, 2013). In this regard, one candidate for the source is the ventral pallium (Puelles et al., 2000; Aboitiz and Zamorano, 2013). This region abuts the pallio-subpallio boundary (PSB), and in mammals functions as a signaling center, secreting morphogens and producing special cell types that regulate brain patterning, including Cajal-Retzius cells (Assimacopoulos et al., 2003; Bielle et al., 2005; Griveau et al., 2010; Teissier et al., 2010; Puelles, 2011). Interestingly, *Dbx1*, the transcription factor expressed specifically in this region of the mouse, is not expressed in the avian corresponding region. Supplementation of *Dbx1* expression in this region of the quail greatly enhances the production of Cajal-Retzius cells (Nomura et al., 2008). These data suggest that this region provides potentially different environments in mammalian and sauropsid lineages (Molnár and Butler, 2002; Molnár et al., 2006; Puelles, 2011). In close proximity to this region is the DVR, which is another distinguishable feature between mammals and sauropsids including both turtles and birds. This anatomical feature could be another candidate for the source of different environmental factors between the two animal lineages.

### POTENTIAL RELATIONSHIPS WITH PREVIOUS HYPOTHESES FOR THE NEOCORTICAL EVOLUTION

Many important hypotheses have been proposed for the evolution of the mammalian neocortex. However, continued debates still rage regarding its homologous counterpart in other animals. Many of the confusions stem from different usages of the term “homology” (Aboitiz, 1999; Molnar and Pollen, 2013; Medina et al., 2013). The term can refer to the same embryonic origin, gene expressions, neuronal connectivities, or other characteristics depending on the proposers. The situation becomes even more complicated because these characteristics belong to biological processes occurring at different levels, and therefore can either be totally independent of, or somewhat related to, each other even for a certain, small, brain domain. We will discuss our observations in relation to the previous hypotheses according to their individual criteria for homology.

#### Embryonic origin

The great diversity of pallial morphologies in animals can be reduced when brains are observed in the very early stages of their development. Indeed, the expression patterns of brain patterning genes in the VZ are highly similar even among distant animal groups, and are therefore highly comparable (Puelles et al., 2000; Murakami et al., 2005; Sugahara et al., 2013). According to this line of analysis, the vertebrate pallium is commonly subdivided into four regions, medial, dorsal, lateral, and ventral (Puelles et al., 2000). Among them, the origin of the mammalian neocortex is attributed to the *Emx*-positive dorsal pallium. Our analyses basically focused on this dorsal pallium compartment. In addition, the medial end of the layer 5 marker-expressing domain such as the mDC in turtles and the APH in chicks probably includes descendants of the medial pallium (Nomura et al., 2008). It is possible that the division between the medial and dorsal pallia is not exclusive. Our previous analysis of neuron migration in the chick pallium did not support the idea of a boundary of cell migration between the two domains (Suzuki et al., 2012). Therefore, a mixed population of medial and dorsal pallial origins seem to constitute the layer 5 marker-expressing domain. On the other hand, tangential mixing of migrating neurons between the mesopallium and nidopallium has not been observed in previous studies (Nomura et al., 2008; Suzuki et al., 2012), suggesting totally distinct origins of the constituents in these two compartments. Because we did not focus on the *Emx*-negative nidopallium in chicks and the DVR in turtles, our results do not exclude the possibility that *Emx*-negative pallial domains contain neurons homologous to the mammalian neocortex judged by different criteria.

#### Gene expression

Gene expression analysis is a powerful approach to detect hidden similarities among regions. Our present study essentially relies on this analysis. Recently, two systematic expression analyses in avian brains have been conducted. One was an *in situ* hybridization study using 52 gene probes in eight avian species (Chen et al., 2013; Jarvis et al., 2013). Interestingly, the authors found similarity in the expression profiles between the HA and nidopallium, and between the HD and the mesopallium, which exhibited a “mirror image organization”. The results of cell migration analyses (Nomura et al., 2008; Suzuki et al., 2012) do not support the common embryonic origin of these mirror-imaged domains. However, the similarity in gene expressions could reflect functional similarities in the domains. Of note is that our gene expression analyses of layer 2/3 markers completely agree with this mirror-image model indicating a similarity between the HD and the mesopallium. The layer 5 marker *Fezf2* also appreciably follows this rule of similar gene expressions in the HA and nidopallium.

The other systemic expression analysis was a comprehensive transcriptomic study that compared expression profiles of about 5000 highly expressed genes between adult chicken pallial domains and adult mouse neocortical layers (Belgard et al., 2013). The study did not detect significant similarity among any combinations of the avian pallial domains and the mammalian neocortical layers, except for a weak similarity between the avian nidopallium and the mammalian layer 4. Because being unable to detect significant similarity does not preclude the possibility of homology, the interpretation of these results is not straightforward. For example, the functional importance of genes for evolution cannot be proportional to their expression levels. By analyzing many abundantly expressed genes together, the real significance of similarity in a minor fraction of genes may be buried. Likewise, mass collection of tissues for RNA sampling could conceal individual neuronal differences, even though most genes are in fact heterogeneously expressed by neurons in each pallial domain, as shown in our results. It will take time and effort to genuinely understand the meaning of comprehensive transcriptome data.

#### Connection patterns

Another important criterion for considering homology of brain regions is neural connection patterns. One such pattern is the “nuclear-to-layered” hypothesis in the avian pallium that proposes one-by-one homologies between the avian pallial subdivisions (nuclei) and the mammalian neocortical layers (Karten, 1991; Jarvis et al., 2005; Jarvis, 2009). Our gene expression data concord well with the proposed avian circuits in the hyperpallium, previously called the wulst, which is analogous to the mammalian somatosensory and primary visual circuits. Under this hypothesis, the HA is regarded as an output component to project to the brainstem (Karten et al., 1973; Reiner and Karten, 1983; Watanabe et al., 1983; Veenman et al., 1995; Wild and Williams, 2000; Suzuki et al., 2012), and indeed this domain expressed the markers for mammalian layer 5 corticofugal neurons (Dugas-Ford et al., 2012; Suzuki et al., 2012). The HD and the mesopallium also expressed the layer 2/3 markers, just as expected (Bradley et al., 1985; Shimizu et al., 1995; Atoji and Wild, 2012; Suzuki et al., 2012). Moreover, other groups have provided evidence that the intermediate region between the layer 5 and 2/3-expressing domains indeed expresses layer 4 markers (Dugas-Ford et al., 2012), and have molecularly supported its function as the major recipient of thalamic axons (Karten et al., 1973). Therefore, the expression data of mammalian layer markers are well correlated with the organization of the proposed homologous connectivities in the avian hyperpallium and mammalian neocortex. Because our analyses focused on the *Emx*-positive dorsal pallium, the proposed homologous component for the auditory circuits in the nidopallium (Wang et al., 2010) is beyond the scope of our results.

The situation seems to slightly differ in turtles. Our preliminary axon labeling suggested the layer 5 marker-expressing domain in the turtle does not make descending connections to the brain stem, but instead the layer 2/3-expressing domain makes descending connections to the thalamus (Bradley et al., 1985; Shimizu et al., 1995; Medina and Reiner, 2000, I. K. S. unpublished observation). Thus, the connection patterns and gene expressions seem to be disconnected in the turtle. This is surprising because many of the layer-specific transcription factors used in this study are well known to regulate connection patterns of cortical neurons in rodents. These functions may not be universal among animal groups, and the downstream genes may be relatively easily changeable in different lineages. Such dissociation of categories for homology in some species may underlie the existence of many different hypotheses for reptilian brain evolution (Aboitiz et al., 2002).

#### Neuronal birth order

Our previous study has added a new aspect to considering homology. The key element for constructing the mammalian neocortex is birth order-dependency in specification of layer-specific neuronal subtypes (Temple, 2001; Okano and Temple, 2009). This is accomplished by each cortical progenitor that follows a stereotyped neurogenetic sequence so as to sequentially produce deep to upper subtypes (Shen et al., 2006; Gaspard et al., 2008). Our results showed that this birth order-dependent mechanism is not specific to mammals (Suzuki et al., 2012), because chick pallial neural progenitors recapitulate this mammalian-type neurogenetic sequence in culture. The *in vivo* chick neural progenitors also follow this mammalian-type sequence in a spatiotemporally restricted manner. Thus, the homology that we have observed in the previous study is likely to represent the birth order similarity of neurons. We suspect the same scenario is applicable to turtles based on the spatiotemporal neurogenetic patterns observed here. This new criterion for homology could be grounded by the shared neurogenetic mechanisms between mammals and sauropsids, and also by the consequent conservation of birth order-dependent regulation of their gene expressions. In this regard, this criterion significantly differed from those that have been conventionally used in comparative anatomical studies, and can thus provide a different dimension to the homology debate.

### POTENTIAL EVOLUTIONARY SCENARIOS OF THE AMNIOTE PALLIUM

**FIGURE 9:**
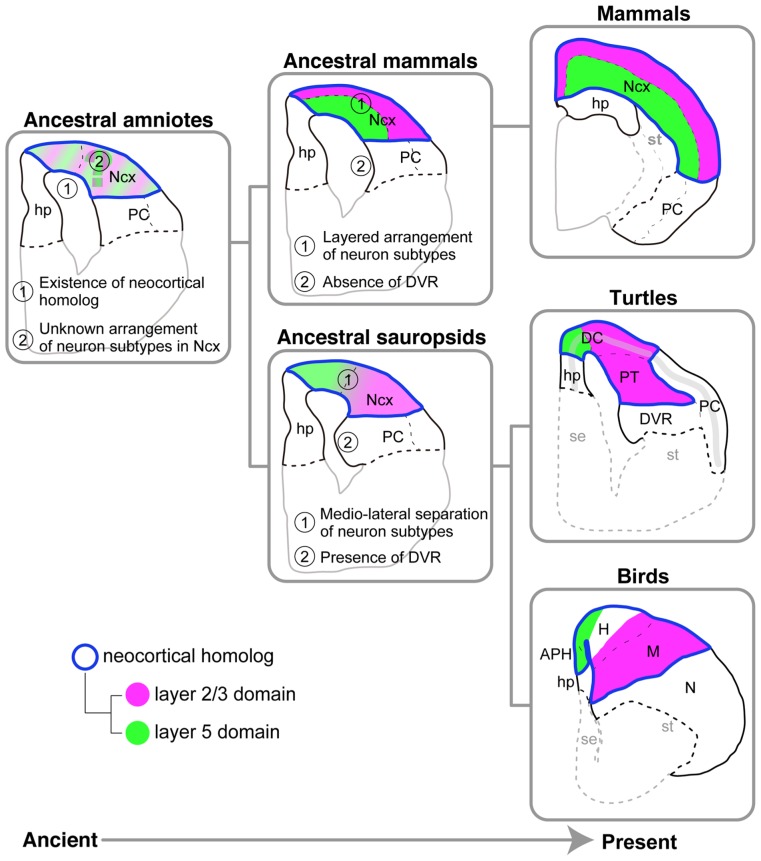
**Models for amniote pallial evolution.** Evolutionary history flows from the ancient on the left side to the present on the right side. The pallia of the three living amniote groups are illustrated on the rightmost side. The hypothetical ancestral states deduced from the living species are illustrated on the center and left sides. The ancestral sauropsids, which are the common ancestors of the turtles and birds, are supposed to have had the following two characteristics shared by living turtles and birds: (1) separation of layer 5 and layer 2/3 subtypes in the medial and lateral domains of the pallium, respectively, and (2) the presence of DVR. The ancestral mammals, which are the common ancestors of all mammalian species, are supposed to have had the following two characteristics shared by all living mammalian species: (1) the layered arrangement of layer-specific neuron subtypes in the neocortex, and (2) absence of the DVR. The ancestral amniotes, which are the common ancestors of all amniote species, are supposed to have had the following two characteristics shared by all living amniotes: (1) existence of a neocortical homolog (Ncx), and (2) existence of layer 5 and layer 2/3 subtype neurons in the neocortical homolog. It remains unclear whether this animal had a layered or mediolaterally separated arrangement of the layer subtypes in the neocortical homolog. Common color codes are used in all illustrations: Neocortical homolog (Ncx, encircled in blue), layer 2/3 neuron homolog (magenta), layer 5 neuron homolog (green). Abbreviations: APH, parahippocampal region; DC, dorsal cortex; DVR, dorsal ventricular ridge; H, hyperpallium; hp, hippocampus (hippocampal homolog); M, mesopallium; N, nidopallium; Ncx, neocortex (neocortical homolog); PC, piriform cortex; PT, pallial thickening; se, septum; st, striatum.

On the basis of the molecular and histological characteristics of the pallium in three distantly related amniotes, the turtles, birds, and mammals, we propose the following hypothetical scenario for pallial evolution (**Figure 9**). The last common ancestor of all three amniote groups already possessed multiple layer-specific neuron subtypes, generated by the conserved neurogenetic program (Suzuki and Hirata, 2012, 2013; Suzuki et al., 2012). The ancient conservation of neocortical subtypes in the stem ancestor of the amniotes is consistent with the preceding models (Karten, 1991, 2013) and distinct from more classical view of sequential addition of new neuronal types during the course of evolution toward the mammals (Marin-padilla, 1978). By spatiotemporally modifying the neurogenetic program, the last common ancestor of sauropsids, which existed around 277 million years ago (Wang et al., 2013), established a pallium in which the layer 5 and 2/3 subtypes were mediolaterally separated. The highly elaborated DVR is another marked feature shared by the turtle and chicken, but not shared by the mammals, and therefore was also present in the pallium of the ancestral sauropsids but not in that of the ancestral mammals (**Figure 9**). Meanwhile, the ancestral mammals developed a layered neocortex by making full use of the same conserved neurogenetic program. The idea of distinct evolutionary origins of the neocortical neuron subtypes and the laminar distribution of them has been already proposed elsewhere (Karten, 2013, 1991). A remaining major question is spatial arrangement of layer-specific subtypes in the pallium of the amniote common ancestor. Future investigation of the amphibian pallium as an outgroup will pave the way for exploring the divergent process of the laminar architecture of the mammalian neocortex and the mediolaterally separated organization of the sauropsid pallium.

## Conflict of Interest Statement

The authors declare that the research was conducted in the absence of any commercial or financial relationships that could be construed as a potential conflict of interest.
